# The effect of CO_2_ enrichment on net photosynthesis of the red alga *Furcellaria lumbricalis* in a brackish water environment

**DOI:** 10.7717/peerj.2505

**Published:** 2016-10-05

**Authors:** Liina Pajusalu, Georg Martin, Tiina Paalme, Arno Põllumäe

**Affiliations:** Department of Marine Biology, Estonian Marine Institute, University of Tartu, Tallinn, Estonia

**Keywords:** Carbon dioxide, Baltic Sea, Net photosynthesis, *Furcellaria lumbricalis*, Marine acidification, Macroalgae

## Abstract

Anthropogenic carbon dioxide (CO_2_) emissions to the atmosphere are causing reduction in the global ocean pH, also known as ocean acidification. This change alters the equilibrium of different forms of dissolved inorganic carbon in seawater that macroalgae use for their photosynthesis. In the Baltic Sea, benthic macroalgae live in a highly variable environment caused by seasonality and rapid changes in meteorological conditions. The effect of increasing water CO_2_ concentration on the net photosynthesis of the red macroalgae *Furcellaria lumbricalis* (Hudson) Lamouroux was tested in short-term mesocosm experiments conducted in Kõiguste Bay (N Gulf of Riga) in June–July 2012 and 2013. Separate mesocosms were maintained at different pCO_2_ levels: ca. 2,000, ca. 1,000 and ca. 200 µatm. In parallel, different environmental factors were measured such as nutrients, light and water temperature. Thus, the current study also investigated whether elevated pCO_2_ and different environmental factors exerted interactive effects on the photosynthetic rate of *F. lumbricalis*. In addition, laboratory experiments were carried out to determine the optimal temperature for photosynthesis of *F. lumbricalis*. The results of our field experiments demonstrated that elevated pCO_2_ levels may remarkably enhance the photosynthetic rate of *F. lumbricalis*. However, the magnitude of this effect is altered by different environmental factors, mainly by changes in water temperature.

## Introduction

Ocean acidification is defined as a reduction in the global ocean pH, caused by the uptake of carbon dioxide (CO_2_) from the atmosphere (Caldeira & Wickett, 2003). In fact, since the Industrial Revolution, the average surface ocean pH has fallen by ∼0.1 units and if global emissions of CO_2_ continue to rise, the pH may decrease 0.2–0.3 units by 2100 (IPCC, 2014). Scenario modelling suggests that the surface water pH in the central Baltic Sea may decrease 0.3–0.4 units by 2100 (Omstedt et al., 2012; Schneider et al., 2015). Moreover, the Baltic Sea is sensitive to an increase in acidity due to low carbonate buffering capacity, which is related to its low salinity, particularly in the northern parts (Omstedt et al., 2015). Any changes in seawater pH also cause shifts in carbonate chemistry: with future increasing CO_2_ concentration and decreasing seawater pH, bicarbonate ion (HCO_3_^−^) will become slightly more available and carbonate ion (CO${}_{3}^{2-}$) less available (Raven et al., 2005). These fundamental changes in carbonate chemistry of seawater due to ocean acidification are predicted to cause extensive changes in marine ecosystems (Doney et al., 2009).

The majority of recent studies have focused mostly on the responses of calcifying macroalgae to the negative effects of elevated partial pressure of carbon dioxide (pCO_2_) (e.g., Hall-Spencer et al., 2008; Jokiel et al., 2008; Kuffner et al., 2008; Martin & Gattuso, 2009; Kroeker et al., 2010; Baggini et al., 2014). On the other hand, studies conducted with non-calcifying macroalgae have shown instead a positive response to elevated pCO_2_ levels, for example, an increased growth rate (Gao et al., 1991; Kübler, Johnston & Raven, 1999; Eklöf et al., 2012) and enhanced photosynthesis (Porzio, Buia & Hall-Spencer, 2011; Pajusalu et al., 2013). A recent study has shown that the interactive effects of elevated pCO_2_ and temperature had a positive effect on the abundance of algal turfs (Connell & Russel, 2010). Thus, it has been noted that future increasing CO_2_ concentrations in seawater may enhance the competitive benefit of non-calcifying over calcifying macroalgal species (Roleda & Hurd, 2012).

Macrophytes are important structural components in the coastal brackish-water Baltic Sea ecosystems (Martin, 2000; Kontula & Haldin, 2012). In recent years, there has been an increasing amount of literature on the effect of ocean acidification on macrophytes in the Baltic Sea. In mesocosm experiments, Graiff et al. (2015) investigated the combined effects of elevated pCO_2_ and temperature on macroalgae *Fucus vesiculosus* in the western Baltic Sea. They found that elevated pCO_2_ in combination with a warming effect appeared to increase the growth of *F. vesiculosus*. Another study on the seagrass *Zostera marina* and macroalgae from the Kattegat region of the Baltic showed substantial effects from warming and small positive effects of acidification on their growth (Eklöf et al., 2012). Likewise, our earlier pilot study with different macroalgal species showed that higher water CO_2_ concentrations increased the photosynthetic rates of the fast-growing filamentous alga *Ulva intestinalis* and the red alga *F. lumbricalis*, while the brown alga *Fucus vesiculosus* did not respond to increased water CO_2_ in the NE Baltic Sea on a short-term basis (Pajusalu et al., 2013). A recent study from the same area focused on the effect of increased CO_2_ concentrations on three species of charophytes: *Chara aspera*, *C. tomentosa* and *C. horrida*. The results of these short-term experiments showed that *C. horrida* and *C. tomentosa* exhibited increased net primary production while the response of *C. aspera* to elevated CO_2_ concentrations was only slight in brackish water (Pajusalu et al., 2015).

Macroalgae responses to ocean acidification depend on other limiting environmental factors such as nutrients, light, and water temperature and their interactions (e.g., Celis-Plá, Hall-Spencer & Horta 2015; Al-Janabi et al. 2016). Water temperature differences in seawater are mainly related to seasonal and latitudinal variations; for example, in the Baltic Sea where the temperature is characterised by high seasonal and annual variations (Feistel, Nausch & Wasmund, 2008). Water temperature is an important factor regulating macroalgae photosynthesis and growth (Lobban & Harrison, 1994; Beer, Björk & Beardall, 2014). However, the combined effects of increasing CO_2_ and water temperature on the photosynthesis of macroalgae are not well understood (Raven et al., 2011). Studies have shown that the interactive effects of water temperature and elevated pCO_2_ synergistically affect photosynthesis in the red algae *Neosiphonia Harveyi* (Olischläger & Wiencke, 2013) and *Chondrus crispus* (Sarker et al., 2013).

The current study focuses on the red macroalga *Furcellaria lumbricalis* (Hudson) J. V. Lamouroux 1813, which is a common species of marine flora in the colder waters of the North Atlantic and Arctic Oceans (Bird, Saunders & McLachlan, 1991). The species is one of the few rhodophytes that predominate all over the brackish Baltic Sea, including the Estonian coastal waters (Nielsen et al., 1995; Martin, 2000; Kontula & Haldin, 2012; Kersen, 2013). However, in the Baltic Sea region *F. lumbricalis* must cope with harsh environmental conditions such as low salinity (Larsen & Sand-Jensen, 2006), eutrophication-related poor underwater light climate (Pawlak, Laamanen & Andersen, 2009), and high epiphytic load (Kersen, Paalme & Treier, 2013). In addition, due to low salinity the red alga *F. lumbricalis* loses its full life cycle in brackish water environments (Kostamo & Mäkinen, 2006). Macroalgae *F. lumbricalis* communities are important habitats for many animal species, providing them food and shelter, and an important spawning substrate for commercial fish (Andrulewicz, Kruk-Dowgiallo & Osowiecki, 2004). Moreover, *F. lumbricalis* is the only economically important red algal species in the Baltic Sea (Martin, Paalme & Torn, 2006). The commercial importance of *F. lumbricalis* is based on the polysaccharides extracted from these red algae (Bird, Saunders & McLachlan, 1991; Tuvikene et al., 2006; Tuvikene et al., 2010).

The main aim of the current study was to detect the effect of elevated water pCO_2_ on the photosynthesis of *Furcellaria lumbricalis* in the NE Baltic Sea under summer conditions. Considering that in the brackish Baltic Sea the environmental conditions in the photic zone have turbulent dynamics caused by seasonality and rapid changes in meteorological conditions (Voipio, 1981; Feistel, Nausch & Wasmund, 2008), the mesocosm experiments were carried out during two different experimental periods (years). Thus, the present study also investigated whether elevated pCO_2_ and different environmental factors exerted interactive effects on the photosynthetic rate of *F. lumbricalis*. Our hypotheses were that the photosynthetic rate of *F. lumbricalis* would benefit from elevated pCO_2_ and that the response would vary depending on surrounding weather conditions. In addition, based on our preliminary results from the mesocosm experiments, laboratory experiments were carried out to determine the optimal temperature for photosynthesis in *F. lumbricalis*.

## Materials & Methods

### Experimental design

**Figure 1:**
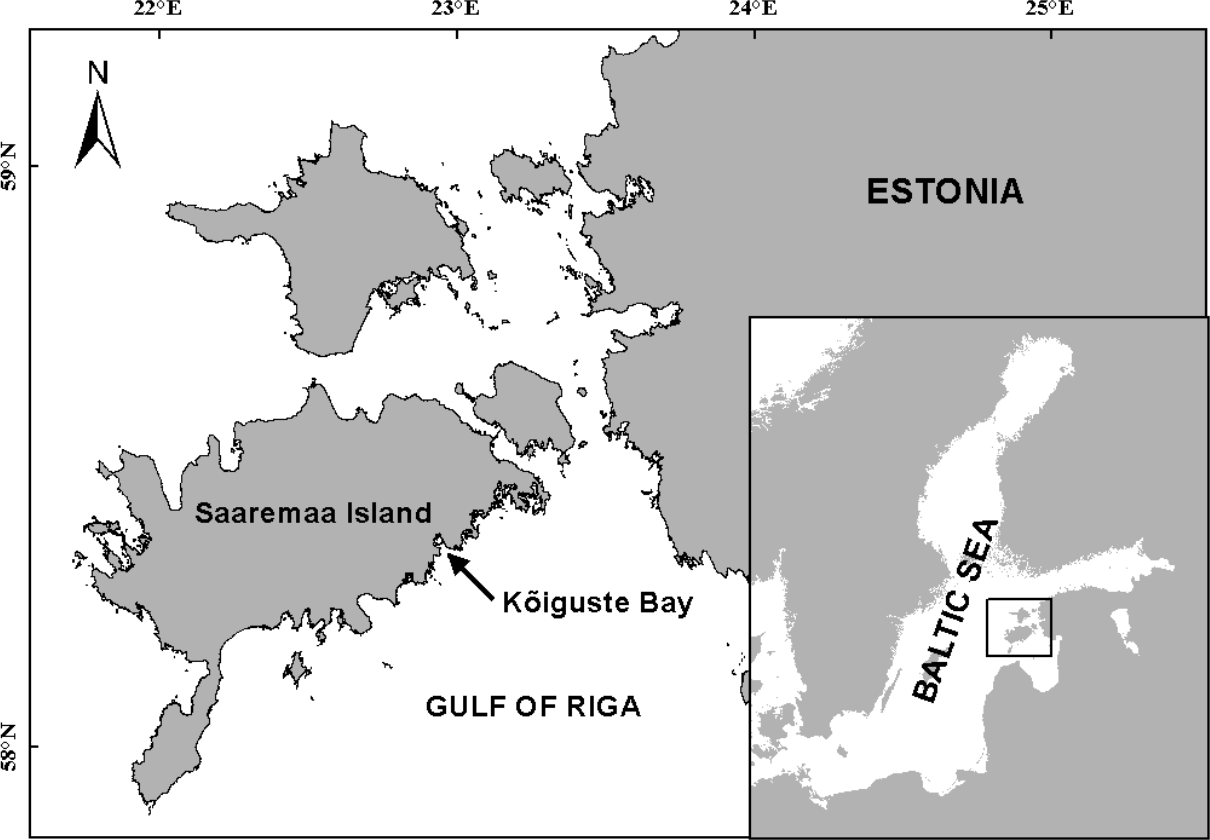
Location of the study area. The arrow shows the location of the experimental site.

The mesocosm experiments were carried out in the shallow semi-enclosed Kõiguste Bay, Gulf of Riga, northern Baltic Sea (58.371°N, 22.980°E) (Fig. 1). The sea area lacks major fresh water inflows, but is affected by nutrient inputs from the moderately eutrophic Gulf of Riga (Astok, Otsmann & Suursaar, 1999; Kotta et al., 2008). The average salinity of the Gulf of Riga varies between 5.0–6.5 PSU (Kotta et al., 2008). The mesocosm experiments were conducted in two experimental periods in 2012 and 2013 (26 June–07 July 2012 and 18 July–27 July 2013). The specimens of *Furcellaria lumbricalis* were collected by SCUBA diving in Kõiguste Bay between depths of 1.5 and 3 m in the site. The collected material was cleaned of all macroscopic epiphytes. The macroalgal material was acclimatised at the experimental site for 24 h prior to the start of the net primary production measurements. About 40 *F. lumbricalis* specimens were incubated unattached at the bottom of each mesocosm. The measurements of photosynthesis were carried out between 10 am and 4 pm.

**Figure 2:**
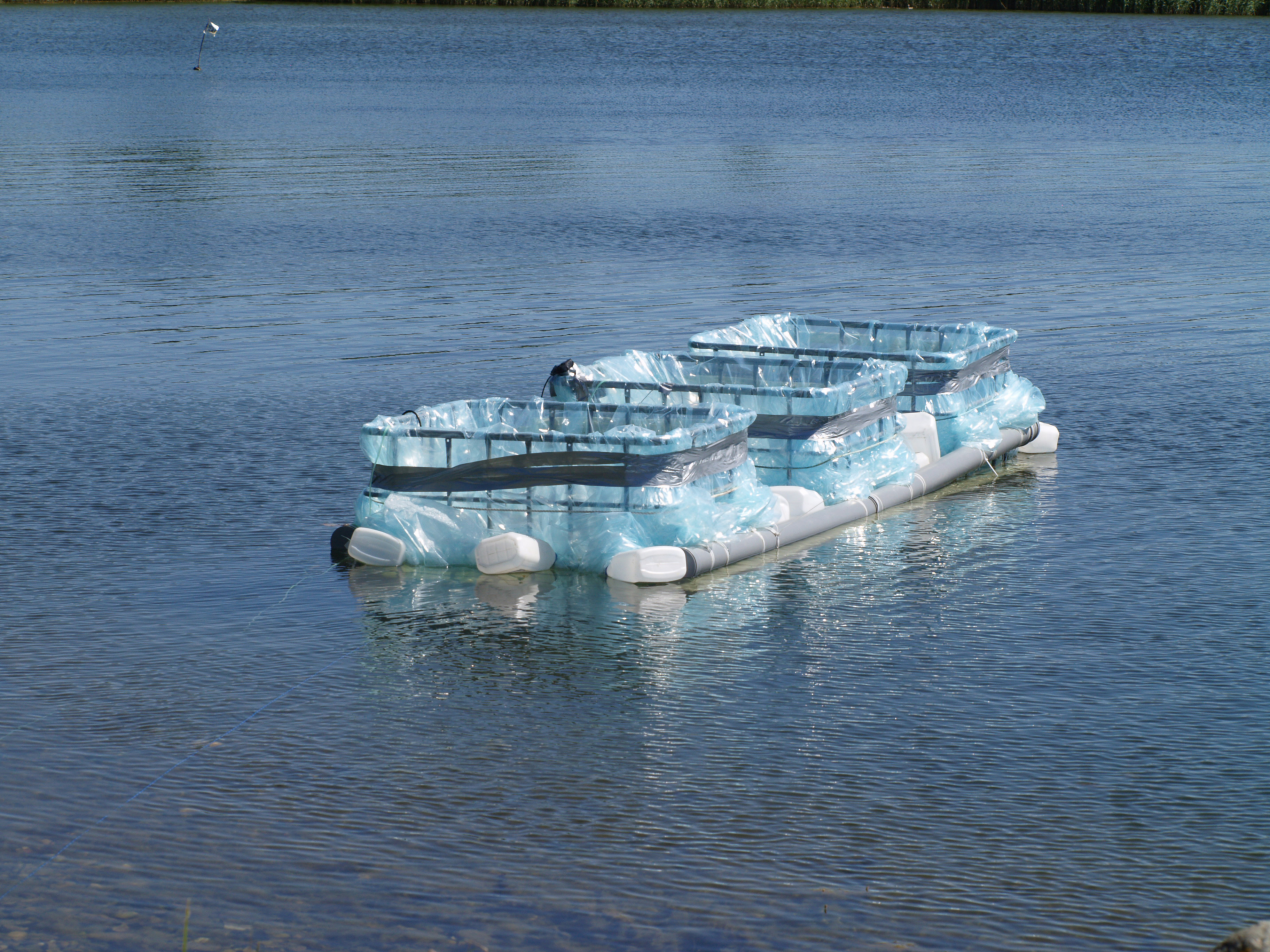
Three plastic bag mesocosms.

The experimental design was identical during both experimental years. Plastic bags (double wall of clear LDPE foil, 175 µm each) externally supported by metal frames were used as mesocosms (Fig. 2). The bags were floating in the sea, fixed to the bottom by anchors. The bags were open on the top, so there was free gas exchange with the atmosphere, but not with the surrounding water. Three plastic bag mesocosms, each with dimensions of 1.2 × 1.0 × 1.5 m, and a volume of 400 l were set up: two mesocosms with elevated pCO_2_ levels ca. 1,000 µatm and ca. 2,000 µatm and one with the untreated level of ca. 200 µatm (control treatment). The pCO_2_ level of 2,000 µatm is much higher than the recommended maximum pCO_2_ level of 1,000 µatm predicted by 2100 for seawater (Barry et al., 2010). In our experiment, the high target pCO_2_ level ca. 2,000 µatm was chosen because natural values of pCO_2_ in Kõiguste Bay measured prior to the start of the mesocosm experiments turned out to be well above the concentration of 1,000 µatm in the summer mornings. Water from the sea area adjacent to the mesocosms incubation site was sieved using a 0.25 mm mesh and used for mesocosms. CO_2_ tanks slowly bubbled food grade carbon dioxide into the water in the mesocosms. The pCO_2_ level was measured using an underwater (sensor) automatic CO_2_ data logger (CONTROS™DETECT 2.0, Germany), connected to a custom-made controller to maintain CO_2_ in mesocosms at required levels. However, due to the response lag of the CO_2_ sensor used (15–20 min), the actual CO_2_ level oscillated by 10% around the level preset by the controller. The pH_NBS_ (National Bureau of Standards scale) values of each treatment were controlled every day before net photosynthesis measurements were taken.

### Environmental variables

In the mesocosm experiments water temperature, oxygen saturation, pH_NBS_ and salinity were measured continuously using a YSI 6600V2 environmental multiprobe (pH electrode YSI 6589FR). Measurements were performed during a full 24-h cycle with a frequency of 30 s. The irradiance at the incubation depths was measured as photosynthetically active radiation (PAR) using a spherical light intensity sensor (Alec Electronics Co Ltd.). Carbonate parameters pCO_2_ and pH_NBS_ and water salinity and temperature were used to calculate total dissolved inorganic carbon (DIC), total alkalinity (A_*T*_), CO${}_{3}^{2-}$ and HCO_3_^−^ using the CO_2_SYS software (Lewis & Wallace, 1998), with carbonate system dissociation constants for estuarine studies (Cai & Wang, 1998). Additionally, in parallel with the photosynthesis measurements, the diurnal fluctuations of water pH, pCO_2_ and oxygen saturation were measured outside the mesocosms at a depth of 0.5 m in the natural shallow water macroalgal habitat. Water samples were taken from the surface in each mesocosms and outside each mesocosm once a day using the standard method ISO 5667-9. The volume of one sample was 250 ml. Samples were frozen immediately in deep-freeze without any liquid until further laboratory analyses using the standard method EN ISO 5667-3. Nutrient concentrations: total nitrogen (TN), total phosphorus (TP), phosphates (P-PO_4_), and nitrites + nitrates (N-NOx) were measured in a laboratory with a continuous flow automated wet chemistry analyzer Skalar SAN^plus^ (Skalar Analytic B.V., De Breda, The Netherlands) using the standard methods EN ISO 11905-1, EN ISO15681-2 and EN ISO 13395.

### Laboratory experiments

The experiments were carried out using the laboratory facilities of the Kõiguste field station of the Estonian Marine Institute, University of Tartu. The specimens of *F. lumbricalis* were collected by SCUBA diving in Kaugatoma Bay (at a depth of 3.5 m) on 30 September 2015. All specimens were placed in coolers containing seawater and transported to the laboratory immediately. In the laboratory, the collected material was cleaned of all macroscopic epiphytes. Specimens of *F. lumbricalis* were acclimated at different water temperatures (5 °C, 10 °C, 15 °C, 20 °C, 25 °C) in 54-litre aquariums (filled with filtered sea water, pCO_2_ ca. 200 µatm) for 7 days before net photosynthesis measurements. The net photosynthetic rate of macroalgae was measured using the oxygen method, described in detail below. The steady temperature was maintained through an active temperature controller (±0.1 °C, AquaMedic cooling units Titan 1500). The light-dark cycle was 12:12 h and during the light cycle under luminophore light the photosynthetically active radiation (PAR) was ca. 200 µmol m^−2^ s^−1^. PAR was measured using a ODYSSEY PAR Logger sensor. The ODYSSEY loggers were calibrated against a LiCor 192 Sensor.

### Measurements of net photosynthesis

For both experiments the photosynthetic rate of *F. lumbricalis* was measured once a day (*n*_day_ = 11 in 2012 and *n*_day_ = 9 in 2013) using the oxygen method. For this procedure about 0.5 g (dry weight, dw) of algal material was incubated in 600 ml glass bottles. During the incubations no stirring inside the bottles was conducted. For field experiments, glass bottles were filled with water from inside the mesocosm and placed horizontally on special transparent trays hanging at a depth of 0.5 m. For laboratory experiments, glass bottles with algal material filled with water from inside aquariums and placed horizontally at the bottom. All incubations with *F. lumbricalis* were performed in triplicate per treatment in mesocosm experiment and in six replicates per treatment in laboratory experiments. Bottles without algae (in triplicate per treatment) served as controls. The dw of the algal material was determined after drying at 60 °C for 48 h. The hourly net primary production (NP) rates (given as mg O_2_ g_*dw*_^−1^ h^−1^) was calculated from the differences in dissolved oxygen concentrations in incubation bottles with and without algal material, measured over the incubation period (ca. 1 h) (Paalme, 2005). The dissolved oxygen concentrations were measured with a Marvet Junior dissolved oxygen meter (MJ2000; Elke Sensor, Tallinn, Estonia) using the standard method EN ISO 5814.

### Statistical analyses

A one-factor permutational multivariate analysis of variance (PERMANOVA) with 9,999 permutations was used to statistically test single and interactive treatment effects on net photosynthetic rate of *F. lumbricalis*: pCO_2_ was used as the fixed factor with 3 levels; photosynthetic radiation (PAR) and water temperature were treated as covariates. Significant effects were explored when necessary with pairwise *post hoc* tests (with 9,999 permutations). The effect of water temperature on the net photosynthetic rate of *F. lumbricalis* in the laboratory experiment was assessed using analysis of variance (ANOVA): temperature as an independent factor with five levels. Tukey’s HSD *post hoc* test was used to find means that were significantly different from each other. Statistical analyses were performed using PERMANOVA+ for PRIMER (PRIMER-E Ltd, Plymouth, UK) and STATISTICA 7.

## Results

### Environmental variables

**Figure 3:**
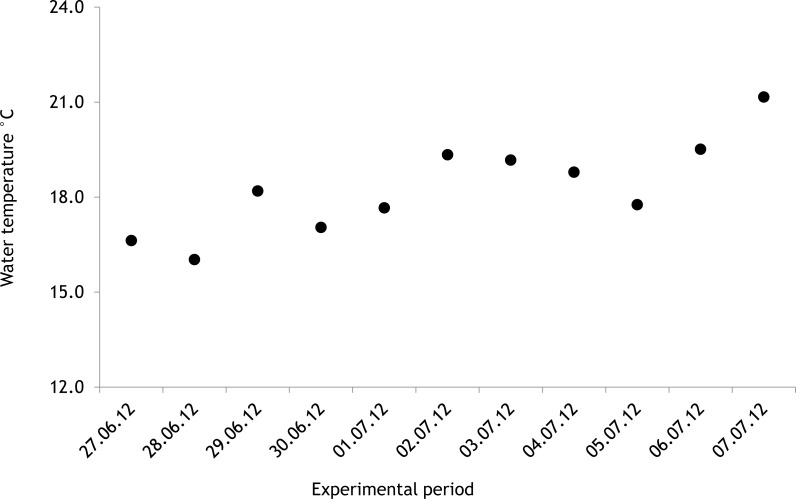
Temporal variation of water temperature during experimental period: 27 June 2012–07 July 2012 (continuous recordings).

**Figure 4:**
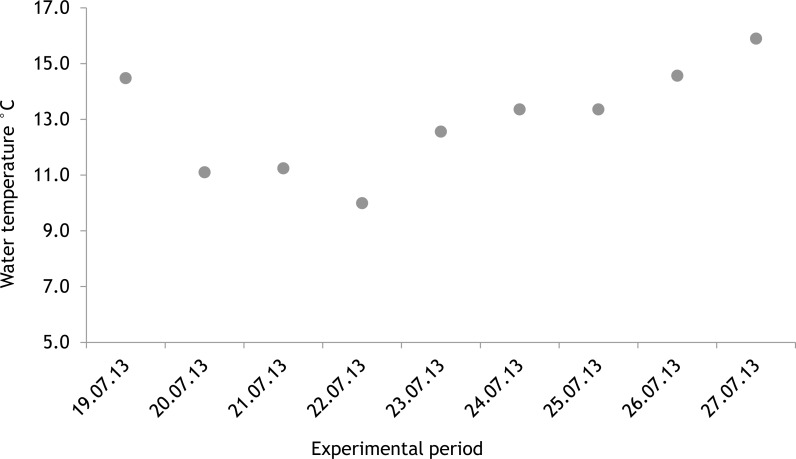
Temporal variation of water temperature during experimental period: 19 July 2013–27 July 2013 (continuous recordings).

**Figure 5:**
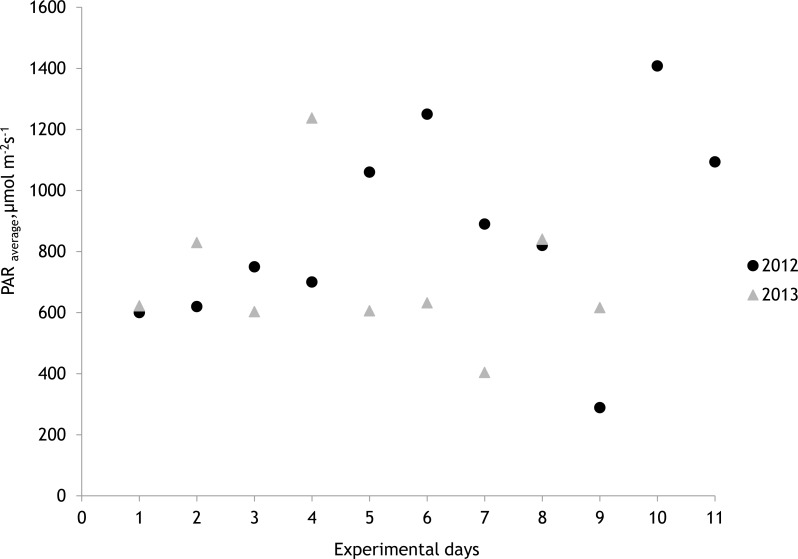
Photosynthetically active radiation (PAR) during the measurements of photosynthesis in 2012 and 2013 measured at a depth of 0.5 m.

The average salinity measured during the experimental period was 5.6 ± 0.02 PSU (±standard error) in 2012 and 5.7 ± 0.01 PSU in 2013. In both years, nutrient concentrations during the experimental period stayed within the limits of typical midsummer conditions (based on databases of the Estonian Marine Institute, University of Tartu) for the area (Table 1). The average water temperature at the experimental site was 18.3 ± 0.45 °C in 2012 and 13.0 ± 0.64 °C in 2013. Temporal variation of water temperature within photosynthesis measurements is presented in Fig. 3 and Fig. 4. PAR is presented as average values for different experimental days and periods in Fig. 5. Parameters of the water carbonate system derived from pCO_2_ and pH_NBS_ for different treatments with pCO_2_ levels in 2012 and 2013 are presented in Tables 2 and 3.

**Figure 6:**
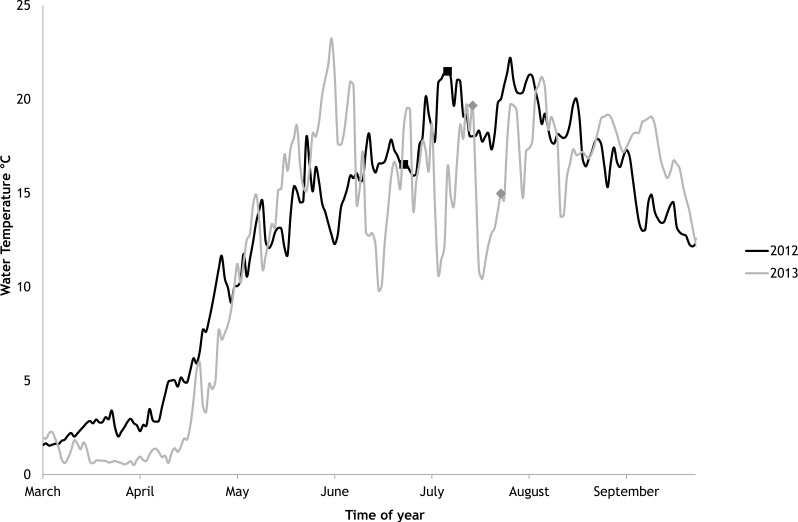
Temporal variations of water temperature during the vegetation periods of 2012 and 2013 (continuous recordings). The markers point to the start and end of experimental periods.

In 2012 the water temperature stayed rather low until July when it reached average summer temperatures and varied during the experiment between 14.9 °C and 22.2 °C. In 2013, the water temperature rose rapidly reaching the season maximum in early June but for the rest of the summer the water temperature was rather low and fluctuated constantly. The experimental variation of water temperature was 8.6 °C to 17.5 °C (Fig. 6).

**Table 1:** Total nitrogen (TN), total phosphorus (TP), phosphates (P-PO_4_), and nitrites + nitrates (N-NO_*x*_) content (mean of the experimental period ± standard error) of the water in mesocosms 2012 (*n* = 33), 2013 (*n* = 27) and of the water outside mesocosm 2012 (*n* = 11), 2013 (*n* = 9).

Experimental period	TN (µmol l^−1^)	TP (µmol l^−1^)	P-PO_4_ (µmol l^−1^)	N-NO_*x*_ (µmol l^−1^)
2012 (water outside mesocosm)	21.7 ± 0.76	1.07 ± 0.05	0.41 ± 0.04	0.43 ± 0.03
2012 (water in mesocosms)	25.1 ± 0.81	1.08 ± 0.03	0.34 ± 0.02	0.30 ± 0.04
2013 (water outside mesocosm)	21.9 ± 1.29	0.77 ± 0.18	0.61 ± 0.14	0.49 ± 0.06
2013 (water in mesocosm)	27.0 ± 1.02	0.68 ± 0.02	0.56 ± 0.02	0.36 ± 0.11

**Table 2:** Parameters of seawater carbonate system in the experiment during the experimental period in 2012. Values were determined from pCO_2_ and pH_NBS_, data are the mean ± standard error of each of the three different pCO_2_ treatments sampled every day before net photosynthesis measurements over the experimental period (*n* = 11).

Treatment pCO_2_	Salinity	T (°C)	pH	pCO_2_ (µatm)	DIC (µmol kg^−1^)	A_*T*_ (µmol kg^−1^)	CO${}_{3}^{2-}$ (µmol kg^−1^)	HCO_3_^−^ (µmol kg^−1^)
200 µatm	5.6 ± 0.02	18.3 ± 0.45	8.91 ± 0.02	198 ± 17.67	4271.6	4832.0	534.4	3729.3
1,000 µatm	5.6 ± 0.02	18.3 ± 0.45	8.04 ± 0.03	1040 ± 32.42	2734.9	2750.8	51.07	2642.4
2,000 µatm	5.6 ± 0.02	18.3 ± 0.45	7.64 ± 0.03	2010 ± 116.	2128.8	2067.0	15.6	2033.1

**Table 3:** Parameters of seawater carbonate system in the experiment during the experimental period in 2013. Values were determined from pCO_2_ and pH_NBS_, data are the mean ± standard error of each of the three different pCO_2_ treatments sampled every day before net photosynthesis measurements over the experimental period (*n* = 9).

Treatment pCO_2_	Salinity	T (°C)	pH	pCO_2_ (µatm)	DIC (µmol kg^−1^)	A_*T*_ (µmol kg^−1^)	CO${}_{3}^{2-}$ (µmol kg^−1^)	HCO_3_^−^ (µmol kg^−1^)
200 µatm	5.7 ± 0.01	13.0 ± 0.64	8.94 ± 0.04	206 ± 16.67	5081.6	5701.5	598.3	4473.6
1,000 µatm	5.7 ± 0.01	13.0 ± 0.64	7.98 ± 0.06	985 ± 61.33	2426.0	2419.0	34.4	2345.5
2,000 µatm	5.7 ± 0.01	13.0 ± 0.64	7.53 ± 0.06	1986 ± 14.56	1779.7	1697.1	8.73	1677.9

### Net photosynthetic rate of *Furcellaria lumbricalis* at different water temperatures

**Figure 7:**
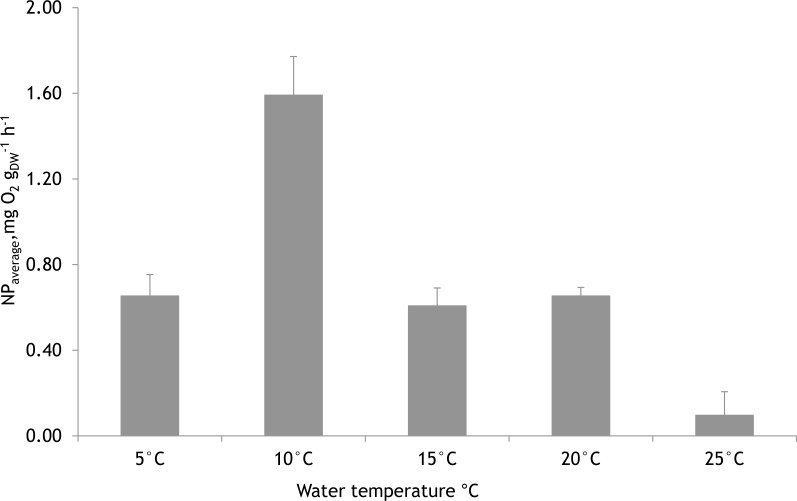
Mean net primary production rates of *Furcellaria lumbricalis* at different water temperatures in the laboratory conditions (PAR ca. 200 µmol m^−2^ s^−1^ and pCO_2_ ca. 200 µatm). Bars represent mean ± standard error, *n* = 30.

Based on laboratory experiments, the water temperature showed a significant effect on the NP rates of *F. lumbricalis* (one-way ANOVA, *F* = 23.11, *p* < 0.05, *n* = 30). The significantly higher average photosynthetic rate of *F. lumbricalis* was measured at 10 °C compared to NP rates at 5 °C, 15 °C, 20 °C and 25 °C. At the same time, according to Tukey’s HSD *post hoc* test there were no remarkable differences in NP rates measured between 5 °C, 15 °C and 20 °C (*p* > 0.9) while the significantly lower average NP rate of *F. lumbricalis* was measured at 25 °C compared to the above-mentioned temperatures (Tukey’s HSD *post hoc* test, *p* < 0.05, Fig. 7).

**Figure 8:**
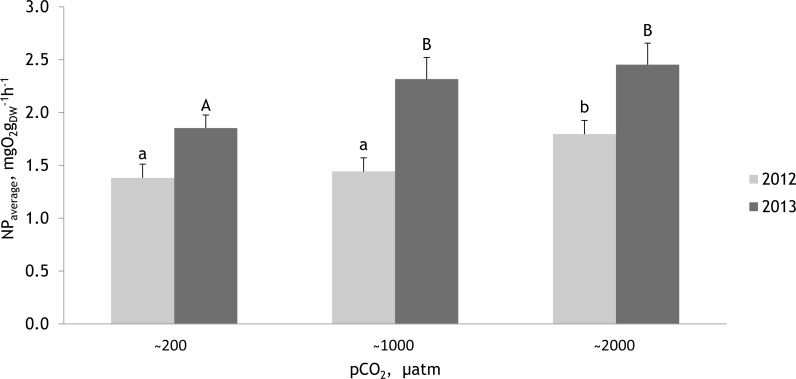
Mean net primary production rates measured within two experimental periods (27 June 2012–07 July 2012 and 19 July 2013–27 July 2013) for the red alga *Furcellaria lumbricalis* at different seawater pCO_2_ levels. Within experimental periods, the same lowercase letters in 2012 and uppercase letters in 2013 indicate no significant differences (PERMANOVA pair-wise test for factor CO_2_). Bars represent mean ± standard error (*n* = 33 in 2012 and *n* = 27 in 2013).

### Net photosynthetic rate of *Furcellaria lumbricalis* at different pCO _2_ levels

The PERMANOVA analysis (conducted separately with the data obtained in 2012 and 2013) showed that the net photosynthetic rate of *F. lumbricalis* varied significantly between treatments at different pCO_2_ levels (Fig. 8; Tables 4 and 5; PERMANOVA: *p* < 0.05). In both experimental periods the hourly net primary production (NP) rates of *F. lumbricalis* showed similar response patterns to elevated CO_2_. The highest NP rates for *F. lumbricalis* were measured under high pCO_2_ values, but also at the intermediate pCO_2_ level the macroalga had higher rates than in the control conditions (Fig. 8A). The highest NP rate was measured under high pCO_2_ level: 3.68 mg O_2_ g_*dw*_^−1^ h^−1^ in 2013 and the lowest NP rate was measured at the intermediate pCO_2_ level: 0.59 mg O_2_ g_*dw*_^−1^ h^−1^ in 2012. Based on a PERMANOVA pair-wise *post hoc* test, the differences in the NP rates of *F. lumbricalis* at the pCO_2_ levels of ∼200 µatm and ∼1,000 µatm were slight but at the pCO_2_ level of ∼2,000 µatm a significantly higher photosynthetic rate was measured compared to the lower levels in 2012 (Fig. 8B). In 2013 the differences in the NP rates of *F. lumbricalis* at the pCO_2_ levels of ∼1,000 µatm and ∼2,000 µatm were slight, but at the pCO_2_ level of ∼200 µatm a significantly lower average photosynthetic rate was measured (PERMANOVA pair-wise *post hoc* test; Fig. 8C). At the highest pCO_2_ level of ∼2,000 µatm the average NP values of *F. lumbricalis* were 1.80 mg O_2_ g_*dw*_^−1^ h^−1^ in 2012 and 2.45 mg O_2_ g_*dw*_^−1^ h^−1^ in 2013. At the intermediate pCO_2_ level the average NP value was 1.44 mg O_2_ g_*dw*_^−1^ h^−1^ in 2012 and 2.32 mg O_2_ g_*dw*_^−1^ h^−1^ in 2013. The lowest average NP values for *F. lumbricalis* were measured in the control conditions: 1.38 mg O_2_ g_*dw*_^−1^ h^−1^ in 2012 and 1.85 mg O_2_ g_*dw*_^−1^ h^−1^ in 2013 (Fig. 8D).

**Table 4:** Results of PERMANOVA analysis on the separate and interactive effects of pCO_2_, PAR and water temperature on the net primary production rate of *Furcellaria lumbricalis* in 2012. Significant effects (*p* < 0.05) are indicated in bold.

Source	DF	MS	Pseudo-F	P(perm)	perms
Water temperature	1	148.63	0.9403	0.3496	9948
PAR	1	2387.3	15.103	**<0.05**	9947
pCO_2_	2	1573.5	99.546	** <0.05**	9943
Water temperature*PAR	1	398.99	25.241	0.0937	9953
Water temperature*pCO_2_	2	2816.6	17.818	**<0.05**	9937
PAR*pCO_2_	2	460.02	29.102	**<0.05**	9945
Water temperature*PAR*pCO_2_	2	426.67	26.992	0.0516	9948
Res	87	158.07			
Total	98				

**Table 5:** Results of PERMANOVA analysis on the separate and interactive effects of pCO_2_, PAR and water temperature on the net primary production rate of *Furcellaria lumbricalis* in 2013. Significant effects (*p* < 0.05) are indicated in bold.

Source	DF	MS	Pseudo-F	P(perm)	perms
Water temperature	1	2688	18.629	**<0.05**	9949
PAR	1	42.684	0.2958	0.6858	9943
pCO_2_	2	1123.9	77.892	** <0.05**	9952
Water temperature*PAR	1	7.628	0.0529	0.9392	9930
Water temperature*pCO_2_	2	310.73	21.535	0.1074	9947
PAR*pCO_2_	2	259.6	17.991	0.1524	9957
Water temperature*PAR*pCO_2_	2	135.78	0.9411	0.4082	9947
Res	69	144.29			
Total	80				

**Figure 9:**
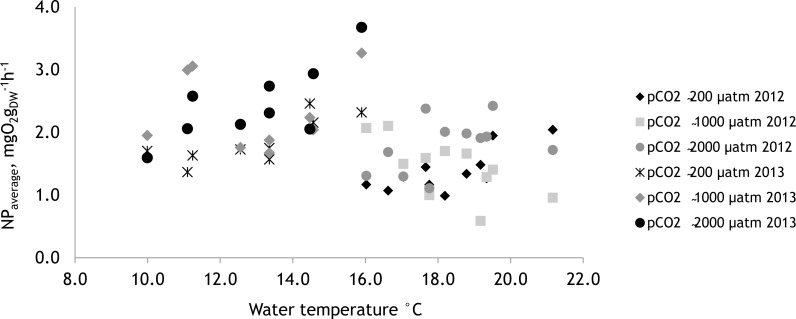
The effect of water temperature on net primary production of *Furcellaria lumbricalis* at different water pCO_2_ levels measured over the two experimental periods (27 June 2012–07 July 12 and 19 July 2013–27 July 2013), mean values, *n* = 3.

The effect of tested environmental factors–water temperature and PAR on NP of *F. lumbricalis*–differed between experimental years. In 2012 the NP rates of *F. lumbricalis* were affected by PAR and the interactive effect of pCO_2_ and PAR as well pCO_2_ and the water temperature (Table 4; PERMANOVA: *p* < 0.05). In 2013, besides the effect of elevated pCO_2_ the NP rates of *F. lumbricalis* were also affected by water temperature (Table 5; PERMANOVA: *p* < 0.05). The higher NP rates of *F. lumbricalis* were measured at the lower water temperatures in 2013 (Fig. 9).

## Discussion

The study demonstrated that elevated water CO_2_ concentrations may enhance the photosynthetic rate of the red macroalga *Furcellaria lumbricalis* in the brackish Baltic Sea. As predicted, based on the results of our earlier pilot study (Pajusalu et al., 2013), the highest photosynthetic rates for *F. lumbricalis* were measured in treatments with high pCO_2_ values. However, some differences between NP rates were found during the two experimental periods. This could be explained by the differences in weather conditions and other environmental factors, first of all by the differences in water temperature. In our experiments higher NP values of *F. lumbricalis* were measured under the lower water temperatures in 2013 (average water temperature 13 °C and average NP 2.21 mgO_2_ g_*dw*_^−1^h^−1^) compared to the values of 2012 (average water temperature 18 °C and average NP 1.54 mgO_2_ g_*dw*_^−1^h^−1^). In 2013 the increase in water temperatures resulted in the increase of NP rates at all pCO_2_ levels, while in 2012 the direction and size of the effect of water temperature on NP rates was dependent on pCO_2_ levels. An increase of water temperature over 19–20 °C showed negative effect on NP rates of *F. lumbricalis* at high pCO_2_ Bird, Chen & McLachlan (1979) indicated that the highest growth rate of *F. lumbricalis* occurs at 15 °C and the growth begins to decline when the temperature rises to 20 °C. The highest NP rates of *F. lumbricalis* in our laboratory experiments were obtained at 10 °C and the lowest at 25 °C. Similarly, during our mesocosm experiments the highest net photosynthetic rates were measured between 10–15 °C, suggesting that *F. lumbricalis* is adapted to water temperatures within this range. Thus, it could be suggested that a future increase in water temperature under climate change may reduce the photosynthetic rate and also alter the response of *F. lumbricalis* to increasing CO_2_ concentrations. Koch et al. (2013) pointed out that the combined effects of increasing CO_2_ and temperature most likely control marine autotroph photosynthetic and growth responses. Olischläger & Wiencke (2013) investigated the combined effects of elevated pCO_2_ and water temperature on the physiological performance of red algae *Neosiphonia harveyi*, from the Helgoland (North Sea) and found that the favourable effects of elevated pCO_2_ were more pronounced at low water temperatures. In the Baltic Sea, where the water temperature is highly dependent on seasonal and annual variations, the effects of rising CO_2_ and water temperature should be observed together.

An important factor that could influence macroalgal responses to ocean acidification is the way carbon is acquired for photosynthesis (Van den Berg et al., 2002; Hepburn et al., 2011). Most of the investigated macroalgae use mainly HCO_3_^−^ from the external medium for photosynthesis (Raven, 2010; Koch et al., 2013; Beer, Björk & Beardall, 2014), which will become slightly more available with the expected increasing CO_2_ content in seawater (Raven et al., 2005). Thus, *F. lumbricalis* which use HCO_3_^−^ for photosynthesis may benefit from a future increase of CO_2_ in seawater (Moulin et al., 2011). However, several investigations showed that macroalgae prefer CO_2_ over HCO_3_^−^ for photosynthesis, and HCO_3_^−^ use can be facultative, i.e., at high CO_2_ concentrations HCO_3_^−^ use is downregulated (Sand-Jensen & Gordon, 1984; Hepburn et al., 2011; Cornwall et al., 2012). Raven et al. (2011) pointed out that this facultative ability of macroalgae to alter the dependence of photosynthesis from HCO_3_^−^ to CO_2_ may provide a competitive advantage at future increasing CO_2_ because of reduced energy requirements for carbon acquisition. High CO_2_ enhances C_3_ photosynthesis because ribulose bisphosphate carboxylase-oxygenase (RUBISCO) has low affinity to CO_2_ and also fixes O_2_, which leads to losses of previously-fixed carbon through the photorespiratory pathway (Bowes & Ogren, 1972; Ehleringer, 2005). Thus, decreases in O_2_:CO_2_ ratios due to increased CO_2_ may explain the increase in net photosynthetic rates. Moreover, compared to other macroalgae phyla, rhodophyte Rubisco has a greater affinity for CO_2_ relative to O_2_, and thus even slight increase in CO_2_ should enhance photosynthesis even without HCO_3_^−^ use or a CCM (Badger et al., 1998; Reinfelder, 2011; Koch et al., 2013).

To define the dissolved carbon concentration saturating the photosynthesis of *F. lumbricalis* further research is needed. Therefore, one of the key questions is whether or not the photosynthesis of macroalgae (including* F. lumbricalis*) is saturated by the seawater DIC under present-day conditions. The carbon-concentrating mechanisms (CCMs) of macroalgae are considered not to be limited by inorganic carbon availability of today’s seawater for photosynthesis (Giordano, Beardall & Raven, 2005). At the same time, Kübler, Johnston & Raven (1999) showed that macroalgae that rely exclusively on CO_2_ diffusion may be carbon limited in the present environmental conditions due to the lower concentrations of CO_2_ compared to HCO_3_^−^. Several studies examining the photosynthesis of seagrasses indicate their limitation by the current seawater DIC, even with their capacity to utilize HCO_3_^−^ (Beer & Koch, 1996; Zimmerman et al., 1997; Beer, Björk & Beardall, 2014). Likewise, our results suggest that the photosynthesis of *F. lumbricalis* (HCO_3_^−^ user) appears to be limited by the current water CO_2_ concentration under summer conditions. Our previous pilot study (Pajusalu et al., 2013) also confirms this suggestion. Consequently, a future rising CO_2_ level could positively affect the photosynthesis of *F. lumbricalis*.

It is well known that coastal eutrophication caused by nutrient over-enrichment such as nitrogen and phosphorus is the main threat in the Baltic Sea. The levels of these nutrients vary greatly between different seasons: during summer and autumn their concentrations are relatively low in a shallow coastal condition (Pawlak, Laamanen & Andersen, 2009). Perennial macroalgae including *F. lumbricalis* are capable of storing nutrient reserves in their thallus from seawater for periods of low nutrient availability (Indergaard & Knutsen, 1990). Therefore, the interaction of an increasing CO_2_ concentration with high nutrient availability in brackish water may increase the photosynthetic rate of F. *lumbricalis* in the north-eastern Baltic Sea. In the Mediterranean, Celis-Plá, Hall-Spencer & Horta (2015) found that the benefits of elevated pCO_2_ on macroalgae were more pronounced when combined with increased nutrients.

Benthic macroalgae live under highly variable pH conditions: daily pH fluctuations may be larger than 1 unit in a shallow-water macrophyte meadow in summer conditions (Pajusalu et al., 2013; Saderne, Fietzek & Herman, 2013; personal measurements). The daily natural variability of brackish-water pH is driven by the direct effects of metabolic activity, such as photosynthesis and respiration in shallow coastal water. These daily pH changes may be of a larger magnitude than the scenario modelling suggests for the surface-water pH decrease in the Baltic Sea by 2100 (Omstedt et al., 2012). Furthermore, Duarte et al. (2010) pointed out the significance of seagrass meadows as a sink for carbon. Photosynthetic processes are likely to buffer ocean acidification in seagrass meadows, but the magnitude of buffering will depend on the metabolic parameters and hydrodynamic processes of each system (Hendriks et al., 2014). As previously mentioned, our results showed that the photosynthesis of *F. lumbricalis* may be carbon limited at the current summer conditions. Therefore, it could be speculated that habitat-forming, widely distributed macroalgal species (including *F. lumbricalis*) may similarly enhance the buffering capacity of benthic macroalgal communities in shallow coastal areas in the Baltic Sea. However, this statement needs further investigation and verification. Furthermore, our present understanding of how daily pH variation could interact with the effects of future increases in seawater acidity is very limited. Cornwall et al. (2013) investigated how the daily fluctuations in the water pH influence the response of a calcifying macroalga *Arthrocardia corymbosa* to the predicted ocean acidification. They found that the response of coralline macroalgae to increased CO_2_ under the fluctuating treatment was stronger than would be predicted under static conditions alone, as the absolute growth rates were even further reduced by the additional negative effects of diurnal fluctuations in pH.

To conclude, the increasing water CO_2_ concentration can be expected to increase the photosynthetic rate of red macroalgae *F. lumbricalis* in the north-eastern Baltic Sea. In the Baltic Sea under the highly variable environmental conditions, the magnitude of this effect will be affected by different environmental factors, mainly by changes in water temperature.

##  Supplemental Information

10.7717/peerj.2505/supp-1 Supplemental Information 1 Raw data for lab experiment

10.7717/peerj.2505/supp-2 Supplemental Information 2 Raw data for field experiments

## Additional Information and Declarations

### Data Availability

The following information was supplied regarding data availability: The raw data has been supplied as Supplementary File.
